# Forced air oscillations – pneumatic capacitance in microfluidic oscillators produces non-linear responses and emergent behaviors†

**DOI:** 10.1039/d4lc00455h

**Published:** 2024-09-09

**Authors:** Sasha Cai Lesher-Pérez, Vishwa Vasani, Jihye So, Shuichi Takayama

**Affiliations:** a Department of Chemical Engineering, University of Michigan Ann Arbor MI USA; b Department of Biomedical Engineering, University of Michigan Ann Arbor MI USA; c George W. Woodruff School of Mechanical Engineering, Georgia Institute of Technology Atlanta GA USA; d The Parker H. Petit Institute for Bioengineering and Bioscience, Georgia Institute of Technology Atlanta GA USA; e Wallace H. Coulter Department of Biomedical Engineering, Georgia Institute of Technology and Emory School of Medicine Atlanta GA USA takayama@gatech.edu

## Abstract

Pneumatic control mechanisms have long been integral to microfluidic systems, primarily using solenoid valves, pressurized gases, and vacuums to direct liquid flow. Despite advancements in liquid-driven self-regulated microfluidic circuits, gas-driven systems leveraging fluid compressibility remain underexplored. This study presents a mathematical and experimental investigation of gas-driven microfluidic circuits, focusing on forced-air oscillators. We derive and validate a first-principles model of microfluidic circuit elements operated under positive pressurization, using a ‘molecular packets’ analogy to elucidate compressibility effects. Our findings reveal that gas compressibility impacts circuit behavior, by acting similar to a large capacitor in the system, which inherently results in longer oscillation periods. As the syringe evacuates, the capacitance decreases, which in turn reduces the oscillation period. Experimental validation of our system demonstrates persistent behavior when using forced air to drive the microfluidic oscillators, this includes assessing devices with various PDMS membrane thicknesses, as well as evaluating device performance under different flow rates and syringe sizes. The forced air oscillators exhibited decreasing periods and capacitance over time, aligning with our theoretical predictions.

## Abstract 2

We develop a model for and explore the effects of compressibility in pneumatically actuated microfluidic circuit elements and experimentally validate the model with a positively pressurized microfluidic air oscillator.

## Graphical abstract

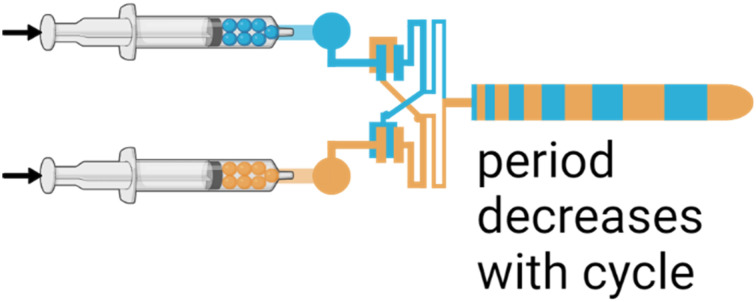

**Tribute to George Whitesides**

In honor of Professor George M Whitesides 85th birthday. Thank you, George, for all your encouragements, red pen inking, thought-provoking questions, and freedom to explore new directions. I am triply blessed by your influence: first, for receiving my PhD from a Whitesides alum, Prof. Chi-Huey Wong, who developed enzymatic organic synthesis with you and significantly expanded it into glycobiology. Second, for the opportunity to have conducted my postdoc with you. Coming from a background in glycochemistry, I initially aimed to focus on molecular self-assembly and multivalency-driven cell interactions. After two years in your lab, however, my approaches to studying cells broadened to encompass microfluidics, soft lithography, and pretty much anything ‘interesting’. Third, for your mentoring of trainees from my lab, including Prof. Bobak Mosadegh—who developed the self-switching microfluidic oscillator in my lab—then helped integrate this technology into soft robotics with you. This article is a further development of the microfluidic oscillator, now driven by air. Please enjoy this article as an extension of your legacy as continued on by my current PhD student Vishwa Vasani, former student and now Professor Sasha Cai Lesher-Pérez, and his student. Happy 85th birthday, George—wishing you many more! Shuichi Takayama

## Introduction

Pneumatic control mechanisms have been a legacy control element within microfluidics, as well as other fluidic systems. Pneumatic controls have primarily relied on the use of solenoid valves connected to vacuum or positive pressurized gas, which in turn directs the movement of liquids by switching gating diaphragm valves, such as the “quake” valve, from a closed to opened-state.^1^ The implementation of integrated logic systems within microfluidic devices over the last two decades has emulated electronic circuits with integrated control units and enabled the generation of complex microfluidic circuits. Pneumatic-regulated logic gates have largely driven this field, utilizing a normally-closed valve to generate a NOT, NOR, and NAND (fundamental Boolean logic) gates.^2,3^ The complexity of these systems has expanded, with different configurations of microfluidic architectures and combinations of the logic gates being leveraged to produce cascading phenomena, such as ring oscillators^4^ and multi-step logic sequences.^5^

While pneumatic control mechanisms have successfully achieved the manipulation of liquid systems,^1,5,6^ a secondary class of self-regulated, semi-autonomous microfluidic circuits were established. These self-regulated circuits are controlled by liquid flow and dynamic pressure changes, and have been demonstrated in constant pressure or constant flow (with variable pressure) configurations.^7–12^

By extension, the use of pneumatic or gas-driven systems should easily translate to driving these self-regulated microfluidic circuits yet has not been fully demonstrated (to the best of our knowledge). Unlike liquid-driven systems, gas-driven systems provide one very palpable yet nuanced characteristic – fluid compressibility. Gas compressibility, while intuitive as a characteristic, adds another layer of complexity and consideration that may result in non-intuitive designing and functionality of gas driven fluidic circuits. Unlike liquid systems which rely on the capacitance inherent to the material properties, such as the distension and deformation of membranes, tubing, *etc.*.., gas provides the dominant capacitive component within the self-regulating fluidic circuit. Defining the role of fluid capacitance in variable pressure systems and other inherent pneumatic properties is key to adequately designing gas-driven fluidic circuits.

In this work, we start with a first-principles model of common microfluidic circuit elements when operated using a compressible fluid under positive pressurization, and then build an example circuit of a self-regulated microfluidic oscillator. Compressibility effects are nuanced and can be difficult to intuit, so in this work, with each description of a circuit element, we attempt to help develop the reader's physical intuition by using a ‘molecular packets’ analogy. In this analogy, each ‘packet’ has the same intensive properties (pressure, temperature, density) as the macroscopic fluid continuum, and contains a fixed number of fluid molecules, but the volume of each packet depends on the local pressure following the ideal gas law. We additionally describe unique properties of these forced-air oscillators observed experimentally due to the compressibility of the fluid, including longer periods, frequency drift and tri-stable oscillations.

## Materials and methods

### Theoretical model

#### Individual circuit elements definition

Calculations and plot generation were done in Python using libraries – numpy, pandas, matplotlib and seaborn. For resistance plots, viscosity of water and air at room temperature were considered. For both gas and liquid, the outlet pressure was assumed to be atmospheric pressure. Representative geometry assumed is 100 μm width and height, and 1 cm length. For valve capacitance, a square cross section was assumed with a length of 500 μm, a depth of 100 μm, and a membrane thickness of 100 μm, with material properties for PDMS from Armani, *et al.*^13^ For syringe capacitance, the dimensions for a 60 mL BD syringe^14^ were used, and the material properties for molded polypropylene^15^ was assumed for the syringe walls. Relative pressure within both the valve and the syringe was assumed to vary from 5 kPa to 10 kPa, which is taken to be the threshold closing and opening pressure respectively for the valve.

#### Full circuit simulation

Differential equations connecting individual components within the microfluidic circuit are derived in ESI.† This system of differential equations was solved numerically, and results were plotted in Python using libraries – numpy, scipy(odeint), matplotlib and seaborn. Equations for pressurization were solved, followed by equations for depressurization, using initial pressure values the same as the final pressure values of the previous iteration. Equations for both the pressurization and depressurization of the valve were numerically integrated in python using the ODEINT function of SciPy library^16^ for 1000 s for each half of the cycle, with a timestep of 1e–3 s, and the results were truncated when the valve pressure reached the threshold opening and closing pressure respectively. Even though we only discuss the valve pressure during the simulation, we simultaneously solve for the syringe pressure because these equations are inter-related. The threshold pressures used in the simulation were obtained from experimental results.

### Experimental data

#### Microfluidic oscillator generation

Microfluidic master molds were fabricated for a final target height of 100 μm. Master molds were generated using SU-8 photolithography techniques as previously described.^17^ The microfluidic oscillator device consists of three polydimethylsiloxane (PDMS) layers assembled as previously described.^8,17^ Briefly, a top and a bottom imprinted PDMS layer are produced from 1 : 10 (curing agent : base) of Sylgard 184 (Ellsworth adhesives) and cured at 60 °C for 4 hours. PDMS membranes were fabricated by spin-coating 1 : 10 PDMS onto glass slides pre-treated with silane. PDMS membranes of two different thicknesses were used in device fabrication: 20 μm and 100 μm. All device fabrication procedures were the same regardless of membrane thickness. PDMS membranes were then cured within a gravity convection oven for 5 min at 120 °C and 10 min at 60 °C. Prior to final assembly, a 2 mm biopsy punch was used to remove PDMS from the inlet and outlet ports of the top device layer. The bottom layer and membrane were first bonded, using plasma oxidation 40 s, 80 W treatment (Covance MP, FemtoScience, Hwaseong-si, Gyeonggi-do, South Korea). After bonding, the bottom layer-membrane assembly was placed in a gravity convection oven at 120 °C for 5 min and at 60 °C for 10 min. Thru-holes were then made in the membrane to allow fluid movement between the top and bottom device layers, using a 350 μm biopsy punch (Ted Pella Inc., Redding, CA, USA). The top layer and membrane-bottom layer assembly were then treated with plasma oxidation. Following treatment, but preceding bonding, the normally closed region of the top layer was “deactivated” by being brought into direct contact with an unoxidized PDMS “stamp”. Once the normally closed valves were deactivated, the layers were bonded, and the assembled device was incubated for 2 min within a gravity convection oven at 120 °C.

#### Microfluidic oscillator testing

Syringe pumps (model KDS220, KD Scientific, Holliston, MA, USA and model Fusion 200, Chemyx, Stafford, TX, USA) were used to provide constant volumetric flow to the device, and 60 mL and 140 mL plastic syringes (Becton, Dickinson and Company, Franklin Lakes, NJ, USA) were used to actuate the fluid (gas) into the system. Microfluidic oscillators were monitored by connecting pressure sensors (Model 142PC05D, Honeywell, NJ, USA) at the device inlets *via* Tygon tubing (Saint-Gobain™ Tygon™ R-3603 Clear Laboratory Tubing, Saint-Gobain Performance Plastics, Akron, OH, USA) to continuously measure source pressure. Source pressure data was collected for both valves to quantify pressure buildup and release corresponding to fluid accumulation and evacuation, similar to work previously described.^17^ Voltage data were collected using LabVIEW and processed, using MATLAB, in part, through the use of the open-source peakdet.^18^

## Results

### Resistance: gas *versus* liquid

The microfluidic oscillator design here is similar to that in Kim *et al.*,^8^ but the analysis carried out in that previous work was limited to systems driven by incompressible fluids such as water or PBS. In contrast, for a gas-driven oscillator, it is important to consider compressibility effects of the working fluid. Similar to the circuit analysis for incompressible fluids, we break down our microfluidic circuit into individual components with electric analogues such as resistance and capacitance, and then work to translate definitions for the circuit elements in gas-driven systems.

For analyzing flow through a straight channel, we use an approach similar to that in Duncan *et al.*^4^ The mathematical details are worked out in SI1.1.1,† but the final results are as follows:

Resistance of a straight channel with circular cross section in a liquid driven system is given by1
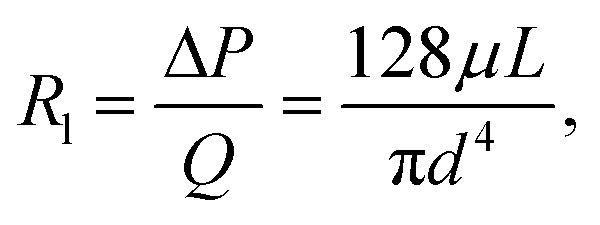
whereas for a gas driven system it is given by2
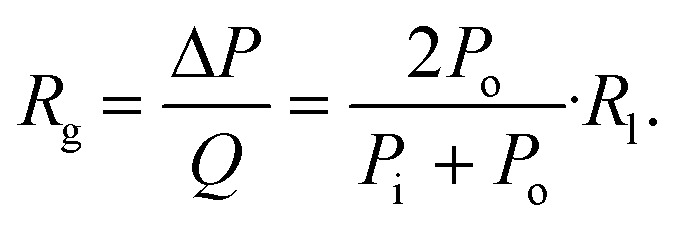
Resistance, *R*, is defined as the ratio of pressure drop Δ*P* across the channel to the flow rate, *Q*, at the outlet. Unlike liquid-driven systems, the resistance for a straight channel with a gas working fluid is not constant for a given geometry of the channel, but instead depends on the pressure values at either end of the channel.

**Fig. 1:**
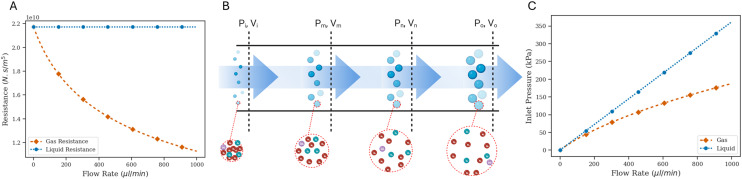
(A) Comparing the resistance of a straight channel for an incompressible liquid and a compressible gas as a function of flow rate. (B) Schematic for the flow of gas through a straight channel. Density varies with pressure; therefore, the volumetric flow rate is variable while the mass flow rate is constant. (C) To drive a higher flow rate through a channel, the marginal increase in inlet pressure required for gas is lower than that for liquid.

Considering a specific example case with a representative straight channel geometry of length 10 mm, width and height 100 μm, and the channels outlet pressure is set to atmospheric pressure, we plot the trend for the liquid resistance (with a hypothetical incompressible liquid with a viscosity equal to that of air) and gas resistance (with compressible air) as a function of outlet flow in Fig. 1A.

Compressible gas flows are inherently non-intuitive, so Fig. 1B and C are included to help develop a physical intuition for the different resistance-to-flow rate relations for incompressible and compressible working fluids. For a compressible working fluid such as air, we define ‘packets’ as arbitrary units of matter containing a constant number of gas molecules, such that each packet has the same macroscopic intensive properties as the bulk properties of the fluid at that location. Following mass conservation for an incompressible liquid's steady flow through a straight channel, the inlet experiences an equal volumetric flow rate as that at the outlet. However, in the case of a compressible gas-driven channel, the volumetric flow rate is lower at the inlet than that at the outlet (Fig. 1B). This is because the fluid needs a negative pressure gradient to drive flow, and for the higher pressure at the inlet compared to outlet, volume per packet is lower at the inlet than that at the outlet. In order to drive the same volumetric flow rate at the outlet, a lower pressure gradient is needed in case of a gas working fluid compared to liquid (Fig. 1C). Since resistance is defined as the ratio of pressure drop to volumetric flow rate (Δ*P*/*Q*) at the outlet, for the same volumetric flow rate, the resistance of a channel with a gas working fluid would be lower than that of a channel with a liquid working fluid.

### Syringe capacitance: gas *versus* liquid

Syringe pumps are commonly used to drive liquid through microfluidic systems, but when these pumps are repurposed for gas-based systems, the flow characteristics differ. Typically, syringe pumps take the desired fluid flow rate as input, and based on the syringe's cross-sectional area, push the syringe plunger with a speed that would produce the desired flow rate. Because liquids are typically incompressible, the rate of reduction of the syringe volume is equal to the volumetric flow rate at the outlet. However, in the case of a gas-filled syringe, the forward motion of the syringe plunger can induce a combination of compression and displacement of the gas.

**Fig. 2:**
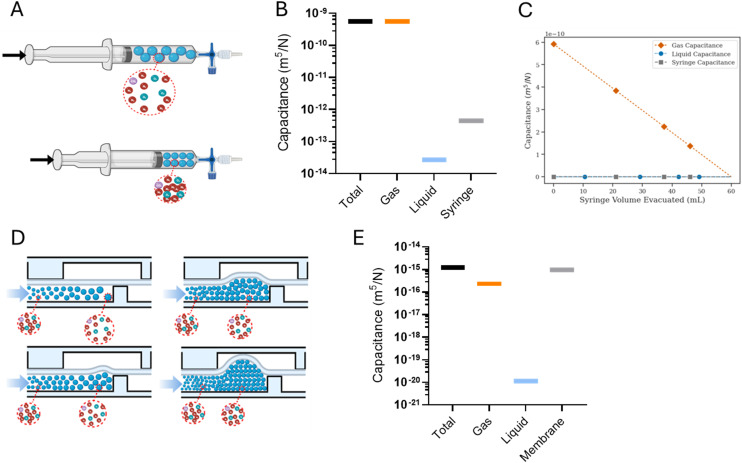
(A) Schematic for the source of capacitance in a syringe. Theoretically, the compressibility of gas and the flexibility of the syringe material both contribute. (B) Total capacitance, and the individual contributions of fluid compressibility and syringe material deformability to it, varying with pressure within the syringe through one pressurization cycle. Data is plotted as boxplots for the capacitance at relative pressures from 5 to 10 kPa, demonstrating no visually identifiable variability of the data. (C) For a syringe filled with gas and for that filled with liquid, variation of the major contributor of total capacitance with changing volume of the syringe. (D) Schematic for pressurization of a microfluidic valve with gas. The compression of gas and the deformation of the membrane can be modeled as capacitive components contributing to the total capacitance of a valve. (E) Plot for the total capacitance of a microfluidic valve, and the individual contributions of fluid compressibility and membrane deformability to it, varying with pressure within the valve through one pressurization cycle. Unlike in the syringe, material deformability is the major contributor of capacitance here. Data is plotted as boxplots for the capacitance at relative pressures from 5 to 10 kPa, demonstrating no visually identifiable variability of the data.

Extending the ‘packets’ analogy to a compressible fluid within a syringe can help intuit its flow characteristics. As the syringe plunger is pushed, and following the subsequent pressurization, each individual fluid packet assumes a volume according to the local pressure, whether it stays within the syringe and compresses, or escapes the syringe and distends (Fig. 2A). Furthermore, fluid pressurization can cause the distension of syringe walls in a manner dependent on the rigidity of syringe material. These dynamics play out under the constraint of mass conservation.

In a theoretical scenario where the syringe and the fluid exhibit ideal characteristics – *i.e.*, perfect rigidity for the syringe and perfect incompressibility for the fluid – the mass of the displaced fluid would exactly equal the mass of the fluid exiting the syringe. Consequently, the input volumetric flow rate set on the syringe pump would be accurately achieved at the syringe outlet.

However, real-world conditions introduce complexities when dealing with compressible fluids and the distention of syringe walls. This leads to a discrepancy between the input volumetric flow rate and the actual volumetric outflow rate. This disparity creates an additional fluid volume stored within the syringe, acting akin to a ‘capacitor’. We define the ratio of this additional volume storage rate to the rate of pressurization resulting from pushing the plunger as the total syringe capacitance.3
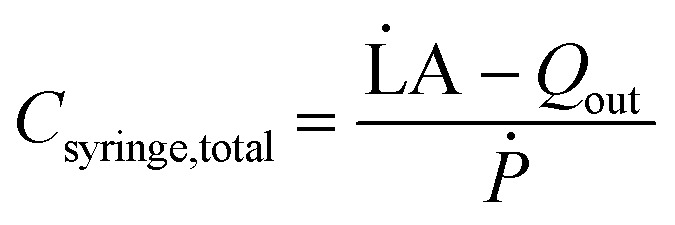
L̇A is the rate of change of syringe volume as a result of syringe plunger being pushed forward by the pump, *Q*_out_ is the volumetric outflow rate at the syringe outlet, and their difference denotes the rate at which additional fluid volume gets stored inside the syringe. Based on the control volume mass conservation analysis carried out^19^ in SI1.1.2,† specifically in the case of either an isothermal or adiabatic process, this additional volume storage relates to the rate of change of pressure as follows:4
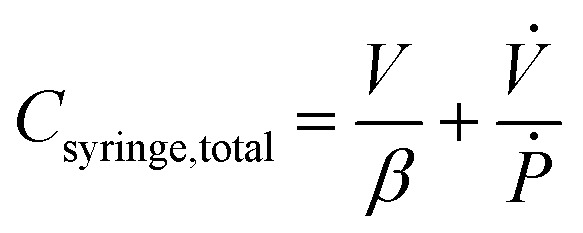
where *β* is the bulk modulus of the fluid within the syringe. The numerical value of *β* will depend on whether the process is modeled as isothermal or adiabatic. 
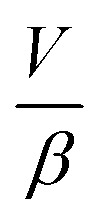
 describes the ability of the fluid contained within the syringe to be compressed, so we define this as the fluid capacitance. 
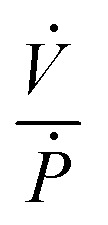
 describes the distention of the syringe walls when being pressurized, so we define this as the syringe wall capacitance and is computed based on mechanics of a pressure vessel.^20^ Total capacitance is then obtained by the linear sum of the capacitance due to deformability of the gas contained within the syringe and the capacitance due to flexibility of syringe walls, and can be represented by the equation

5 *C*_total_ = *C*_fluid_ + *C*_wall_.

The total, as well as the fluid and the syringe capacitances are computed for a full 60 mL BD syringe within one pressurization–depressurization cycle (Fig. 2B). For a gas-filled syringe, the major contribution towards total capacitance comes from fluid capacitance owing to gas compressibility. On the other hand, for a liquid-filled syringe, both the syringe capacitance and the liquid capacitance are orders of magnitude lower than the gas capacitance, and consequently the total syringe capacitance is also lower. As the syringe empties (*i.e.*, syringe volume reduces), the fluid and the material capacitances also reduce (Fig. 2C and S2 and S6C†). This reduction in total capacitance can give rise to time-varying behaviors in a fixed microfluidic circuit with fixed input flow rates.

### Valve capacitance: gas *versus* liquid

The diaphragm valve is a commonly used microfluidic capacitive element. Control volume analysis similar to the one in the case of syringe capacitance can be carried out for a positively pressurized, normally closed diaphragm valve (SI1.1.3†). ‘Valve capacitance’ is defined as the ratio of volumetric flow rate into the valve (*Q*_in_) to the rate of pressurization of the gas within the valve chamber (*Ṗ*)6
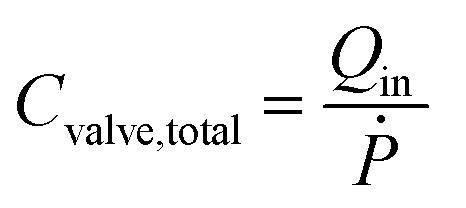
and the resulting equation for valve capacitance is given by7
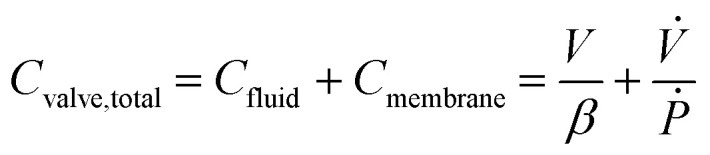
where *V* is the volume enclosed by the membrane valve, *β* is the bulk modulus of the fluid, 
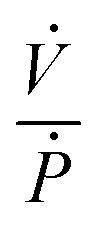
 is the ratio of the rate of change of valve volume due to membrane distension to the rate of change of pressure. Note that the valve capacitance definition is independent of membrane permeability to air and only depends on the material properties of the fluid and the membrane. *Q*_in_ is the net volumetric flow rate into the valve chamber, which can include the flow rate out of the valve through the membrane (*i.e.*, permeability) subtracted from flow rate into the valve at the chamber entrance to give *Q*_in_. Total capacitance of the valve is, like in case of syringe, a linear sum of capacitance due to fluid compressibility 
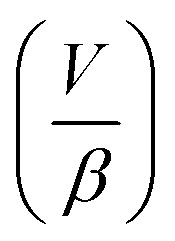
 and that due to membrane deformability 
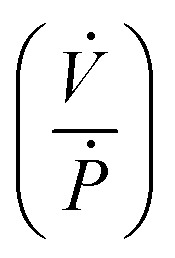
. Extending the packets analogy to diaphragm valves, the membrane capacitance arises because gas pressurization causes the membrane to deform, allowing more ‘packets’ to fit inside the valve chamber, and the fluid capacitance arises because each packet shrinks under pressurization such that more packets can fit inside the valve chamber (Fig. 2D).

For the example valve considered here with known PDMS membrane mechanical properties, the majority contribution to the total valve capacitance comes from membrane deformability (Fig. 2E). The flow characteristic of a positively pressurized valve is minimally affected by the driving fluid, in sharp contrast to a syringe.

### Gas-driven microfluidic oscillator circuit simulation

**Fig. 3:**
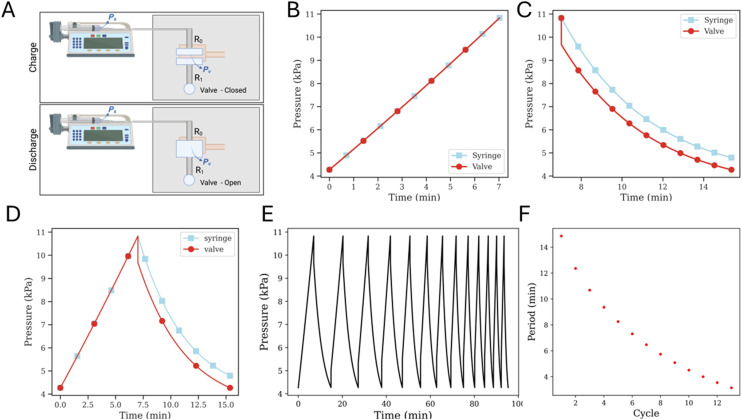
(A) Oscillator full circuit schematic. (B) Single pressurization cycle. (C) Single depressurization cycle. (D) One pressurization–depressurization cycle. (E) Pressure trace for one of the valves in the oscillator system over 13 pressurization–depressurization cycles. (F) Time period over 13 oscillations.

**Fig. 4:**
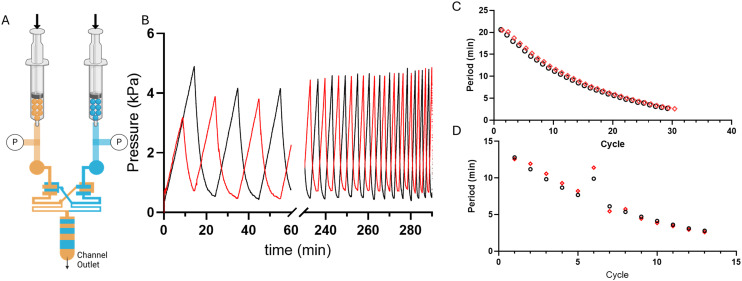
Forced-air oscillators have decreasing periods over time as the syringes' fluid volumes reduce and the syringe pump continues to run. Even though the oscillation period decreases, the oscillatory behavior is persistent. A) A representative schematic of a microfluidic oscillator being driven by gas packets. Two pressure sensors, the circles with (*P*), upstream of the microfluidic oscillator record the pressure profiles of each fluid line. B) A 20 μm membrane oscillator system being driven by two 60 mL syringes at a 200 μL min^−1^ flow rate. Graphed pressure data is split to demonstrate the stark difference in period over the first 60 minutes compared to the (approximately) final 60 minute at the end of the run. C) Period decreases with each oscillation cycle for the 20 μm membrane oscillator and D) a 100 μm membrane oscillator, which was run at 500 μL min^−1^.

Simulating the full oscillator circuit with modified governing equations for a gas working fluid is an involved process, so, in order to make the simulations more tractable, some simplifying assumptions need to be made. The interaction between valves is modeled by assuming a higher threshold opening pressure compared to the threshold closing pressure, and the respective threshold pressure values are obtained from experimental results. Once the valve opens and undergoes depressurization, the outflow splits into two channels – one that vents directly to atmospheric pressure through the proximal outlet, and another that connects to the (non-pressurized) underside of the adjoining valve through a low resistance channel (Fig. 4A). This is difficult to model, so we make the simplifying assumption that when the valve is depressurizing after opening, the branch connecting to the adjoining valve (*i.e.*, the gate terminal for the opposite valve) does not participate in depressurization, and instead it simply presents a high pressure to the gate terminal of the opposite valve, thereby holding it in an ‘off’ position. Then, the valve effectively discharges through the serpentine drain channel, and the circuit simplifies to that as shown in Fig. 3A.

The pressure–volume relations for the syringe, straight channel and valve as described are used as governing equations for each corresponding section within the circuit, and the appropriate boundary conditions are defined at the interfaces of these elements such that the pressure and the flow rate continuity is maintained (SI1.2.1 and 1.2.2†). With this model, the pressurization cycle arises from a continued syringe plunger push until the valve pressure hits the threshold opening pressure. This is followed by the depressurization cycle, which continues until the valve pressure drops down to the threshold closing pressure, and the elasticity of the membrane causes it to relax back to a closed configuration. While the serpentine drain channel has a relatively high resistance due to the geometry (*i.e.*, relatively small cross-sectional area and is a long channel), the connection to the gate region under the opposite valve decreases the downstream resistance. The large dead volume (gate region of the other normally-closed valve) may allow the pressurized valve to discharge without a large change in pressure. When the downstream resistance is changed to that of the channel connecting the two valves, *i.e.* to the same order of magnitude as the upstream resistance, valve discharge proceeds successfully (SI2.2†). For all subsequent simulation results, this modified downstream resistance is used. We also tested the impact of the membrane permeability of gas in our model and find that the results do not change significantly (SI3†). So, for all subsequent simulations, we assume that permeability is negligible.

Simulations for multiple oscillation cycles are carried out as the syringe empties, and correspondingly the system capacitance is found to decrease. The system capacitance is defined in the same way as for experimental results. It is found that the simulation results match experimental results well – with an increasing number of cycles, the period for each oscillation decreases (Fig. 3F and 4C and D).

### Syringe evacuation drives period and capacitance shifts

**Fig. 5:**
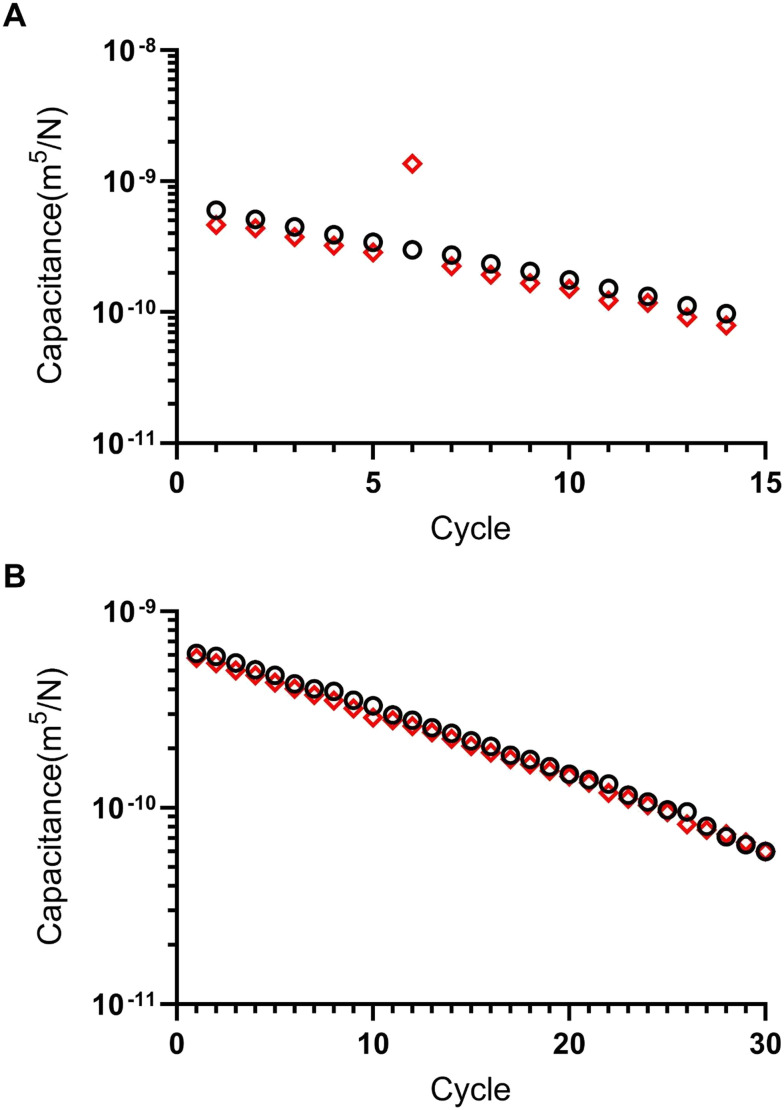
Forced-air oscillator capacitance drops as the syringe fluid volume is evacuated. As the syringe pump runs, and the syringe fluid volume reduces, the total (syringe and fluid) capacitance drops over time, or as presented here after each cycle of oscillations. A) 100 μm thick membrane and B) 20 μm membrane oscillator system. Oscillators were run at 500 and 200 μL min^−1^ respectively and a 60 mL syringe.

Microfluidic oscillators were assessed to determine if gas filled syringes could actuate these microfluidic devices and indeed maintain an oscillatory response. Syringe pumps were set at constant flow rates, resulting in increased pressure and forced air actuating the oscillators similarly to previously described liquid driven systems.^8,17,21,22^ Source pressure (Ps) measurements, taken from the tubing between the syringes and microfluidic devices, demonstrated oscillatory pressure profiles between the two fluidic lines. Uniquely, these forced air oscillators demonstrated a variable, decreasing period (Fig. 4B–D) and capacitance (Fig. 5) throughout a continuous run. Our simulation and theoretical data discussed above aligned well with our experimental results and demonstrated similar behavior. To further validate the occurrence of these forced air oscillations and the intrinsic period reduction and decreasing capacitance observed, we assessed multiple PDMS membrane thicknesses (*i.e.*, 20, 70, 100 μm), syringe flow rates (*i.e.*, 50–1000 μL min^−1^), as well as different syringe sizes (*i.e.*, 60 and 140 mL), to ensure these responses were globally persistent so long as oscillatory behavior was achieved (data presented in ESI†). While our evaluations were in no way exhaustive, we did identify consistently decreasing periods and capacitance as the devices ran and the volume in the syringes was reduced.

The impact of a larger external capacitance (*i.e.*, not the internal valve capacitance) has previously been demonstrated with liquid systems,^22^ where larger external capacitance or total capacitance of the system results in a higher period. In our system we similarly observed increased capacitance as a function of the remaining fluid volume in the syringe, or total volume when comparing two oscillators run with 60 mL *vs.* 140 mL syringes (ESI†).

### Pneumatic systems achieve a unique tri-stable oscillation

**Fig. 6:**
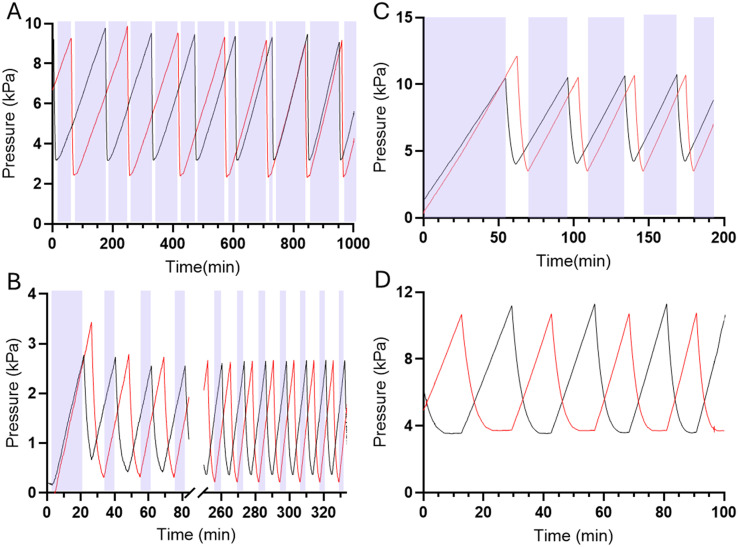
Forced-air oscillators demonstrates a tri-stable oscillation at low flow rates, oscillating between an on state for 1 fluid line (valve 1 open), an on-state for fluid line 2 (valve 2 open), and an off-state, where both valves are closed. In a tri-stable oscillation there are gaps between the on-states, represented in the above images by the purple block on the graphs. Tri-stable oscillations were observed in a (A) 20 μm thick membrane device run at 50 μL min^−1^ (with 60 mL syringes), (B) 20 μm thick membrane device run at 100 μL min^−1^ (with 60 mL syringes), (C) 100 μm thick membrane device run at 200 μL min^−1^ (with 140 mL syringes). There were no tri-stable oscillations in a (D) 100 μm thick membrane device run at 500 μL min^−1^ (with 140 mL syringes) and cycling only between two on-states which is indicated with no purple blocks.

In the forced-air microfluidic oscillators we observed a new operating mode, a tri-stable oscillation, where the system oscillates between two on-states, and one off-state (Fig. 6A and B). In the tri-stable oscillation, forced-air microfluidic oscillators cycle between a state of one valve being open, the other valve opening, and both valves being closed as the pneumatic pressure builds up in each fluid line. The occurrence of the tri-stable oscillation was only observed when operating at lower flow rates (<200 μL min^−1^). We consider that the pressure maintained once the valve opens at these lower flow rates is insufficient to maintain the valve in an open state.

In contrast, liquid-driven microfluidic oscillators have only been reported to cycle between two distinct on-states, where the opening of one valve is diametrically opposed to the closing of the other valve, resulting in one of the two fluids always flowing out of the microfluidic device outlet. Similar to liquid-driven oscillators, higher flow rate forced-air oscillators demonstrate similar pressure profiles of liquid-driven oscillators where the local maxima of one valve's pressure profile aligns with the local minima of the other valve's pressure profile (Fig. 6C).

Previous characterization of the valve closing in liquid-driven fluidic circuits provides us with these insights of why a pneumatic system may achieve this tri-stable oscillation in specific operating conditions.^23^ In a liquid-driven circuit, the closing threshold pressure, *i.e.*, the difference between the source pressure flowing into the valve and the gate pressure underneath the membrane must be negative. These results indicate that pressure in the gate had to exert enough force to evacuate the liquid between the normally closed valve and the membrane to ensure closing of the valve. However, in the case of a pneumatic system, evacuating a gas between the normally closed valve seat and the membrane would require less pressure to eliminate the fluid between the normally-closed valve and membrane. Furthermore, the force exerted by the gas must be sufficient to maintain the membrane in a deformed open position, if the source pressure in the valve becomes sufficiently low, the membrane would return to a normally closed position.

## Conclusion/discussion

In order to design and build sophisticated microfluidic circuits, a clear mathematical description of circuit elements is critical.^5,11,12,21^ The liquid-based microfluidic oscillator first demonstrated by Mosadegh *et al.* applies the electric circuit analogy often used with microfluidic circuits when using incompressible working fluids.^7^ In Lesher-Pérez *et al.*, the same electric circuit analogy allows the understanding of coupling between microfluidic oscillators for emergence of synchronization.^21^ The extension of this mathematical treatment of circuit elements for a compressible working fluid is not straightforward and as such has been attempted in Duncan *et al.* for vacuum-driven circuits.^4^ To our knowledge, the extension to positive pressure-driven circuits has not been attempted yet and can be expected to involve several additional nuances.

Our presented work focused on expanding the electrical analogy for gas-driven microfluidic systems. Specifically, we focused on establishing the contribution of fluid compressibility along with material deformability to the capacitance of a microfluidic element. We found that gas compressibility was the dominant contributor towards syringe capacitance, while the deformability of the membrane was a stronger contributor to microfluidic valve capacitance. We also defined resistance in the context of gas flows and found that it decreased with increasing local flow rate. The updated definitions of microfluidic circuit elements were then applied to full circuit simulations and were found to predict a range of distinctive behaviors specific to a gas-based oscillator system such as higher time period, frequency drift and system capacitance drift.

We then demonstrated a functional self-regulated pneumatic oscillator powered by forced air actuated by syringe pumps. Distinct to previously reported constant flow-driven microfluidic oscillators reliant on liquid inflow, our work here specifically evaluates the contribution and impact of the fluid compressibility in these microfluidic oscillators, which has yet to be explored and described. As a result of the nuanced differences from liquid-based systems, the forced air oscillator showed a range of distinctive behaviors, such as longer oscillation periods, frequency drift and tri-stable oscillations, as predicted by the model.

While broadly applicable, one key area that we see clear potential overlap is in soft robots/robotics. We envision this work has implications for gas-producing chemical reactions, as a feasible mechanism that can provide variable positive pressure profiles to drive these embedded pneumatic circuits.^24^ Furthermore, with the increasing ease and feasibility for constructing integrated microfluidic circuits through 3D printing,^1,25,26^ and 3D printing of soft robots^27–29^ we believe the prevalence and implementation of complex function in soft-robotics and microfluidic system with electronic analogous fluidic control and responsive systems will continue to grow.^30^ Soft robotic actuators when connected in parallel to the circuit upstream of the valve can be expected to add capacitance, reducing the oscillation frequency, whereas actuators connected in series downstream of the valve can be expected to add resistance which may prevent valve from discharging. Downstream actuators having low capacitance can help the valve to discharge quickly without directly venting to atmospheric pressure. Considerations like these, aided by quantitative methods as described in this work to define the compressibility and corresponding control downstream motile elements, such as those present in soft robots,^27^ can help define design criteria for robust forced air actuated systems.

## Data availability

The code for the oscillator model can be found at https://github.com/vishwa-vasani/air-oscillator. Additionally, experimental pressure profile data collected in Open Science Framework, https://osf.io/qjuc5.

## Conflicts of interest

There are no conflicts to declare.

## Supplementary Material

LC-024-D4LC00455H-s001
