# Supplementary material for: Forced air oscillations – pneumatic capacitance in microfluidic oscillators produces non-linear responses and emergent behaviors
Source: Lab Chip. 2024 Sep 9;24(20):4798–807. doi: 10.1039/d4lc00455h (PMC11409223; doi:10.1039/d4lc00455h)
Supplement: LC-024-D4LC00455H-s001 [file LC-024-D4LC00455H-s001.pdf]

Supplementary Information (SI) for Lab on a Chip.
This journal is © The Royal Society of Chemistry 2024

S1 Theoretical Model

In this section, equations for straight channel resistance, syringe capacitance and valve capacitance are derived. Then, full circuit equations connecting the individual circuit elements are derived.

S1.1 Defining Individual Circuit Elements

S1.1.1 Straight Channel Resistance

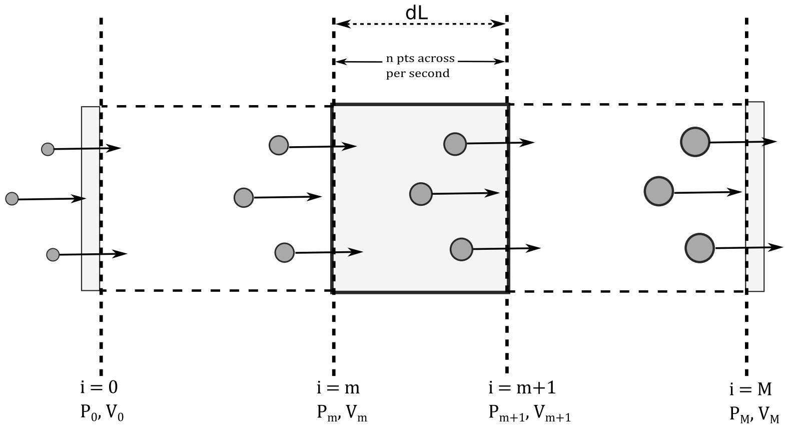

Figure S1: Straight channel resistance schematic. Filled circles are 'packets', which are arbitrary volumes of the fluid containing a fixed number of fluid molecules. The size of the circle corresponds to the volume of the packet at that location.

We follow a procedure similar to that in [1]. We assume that in every infinitesimal section of the channel, flow can be modeled as incompressible, and therefore follows the Poiseuille equation. While compressibility plays a role over a large channel length, we assume that in infinitesimal sections the pressure does not change appreciably and therefore the fluid is not differentially compressed on either end of the section. Then, the pressure-flow rate equation across any arbitrary m^{th} infinitesimal section of the channel follows the relation:

$$-(P_{m+1} - P_m) = Q_{av,m} \times R_{dL} \tag{1}$$

where P_{m+1} and P_m are pressures at downstream and upstream ends of the section respectively, $Q_{av,m}$ is the average flow rate through the section, R_{dL} is the resistance of the channel if an incompressible liquid was flowing through it, and is given by $R_l = \frac{12\mu dL}{wh^3}$ Here, μ is the viscosity of the fluid, dL is the length of the infinitesimal section, w is the width of the channel and h is the height of the channel with a rectangular cross section.

Following the ideal gas law, we have $PV = nRT$, where n is number of moles of the fluid, R is the ideal gas constant, T is the absolute temperature. We assume that there is no accumulation of fluid in the channel, so, following mass conservation, we would have the same number of moles of the fluid passing through each section in the channel. Then, $P_0 \cdot Q_0 = P_m \cdot Q_m = P_{m+1} \cdot Q_{m+1} =$

$P_M \cdot Q_M$, where Q_i is the volumetric flow rate across any i^{th} interface. Then, $Q_i = P_0 \cdot Q_0 / P_i$, and eq.1 then simplifies to the differential equation:

$$-P\frac{dP}{dL} = P_0 Q_0 \cdot \frac{12\mu}{wh^3} \tag{2}$$

Solving eq. 2 across the channel we get

$$\frac{P_0^2 - P_M^2}{2} = P_M Q_M \frac{12\mu L_{channel}}{wh^3} = P_M Q_M R_l \tag{3}$$

Where $L_{channel}$ is the total length of the channel, R_l is the resistance of the channel if an incompressible fluid with the same viscosity was passed through the channel, and only depends on the dimensions of the channel and viscosity of the fluid. For a compressible fluid, the viscosity can change based on the local pressure, but within the range of pressure used in our system, at room temperature, this change is negligible.

Since $P_0^2 - P_M^2$ can also be written as $(P_0 + P_M) \times (P_0 - P_M)$, eq. 3 simplifies to give R_g, defined as the ratio of the pressure drop across the resistor to the flow rate (at the outlet), according to the traditional definition of resistance:

$$R_g = \frac{P_0 - P_M}{Q_M} = \frac{2P_M}{P_0 + P_M} R_l \tag{4}$$

S1.1.2 Syringe Capacitance

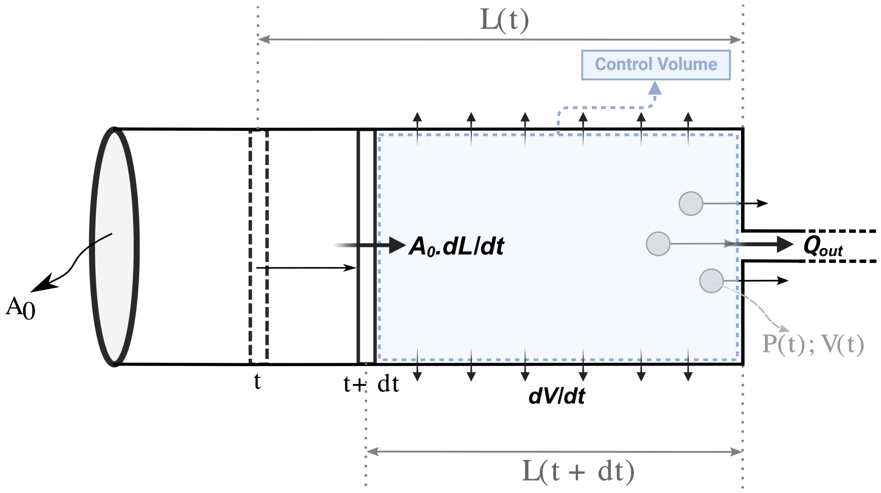

Figure S2: Syringe capacitance schematic. As the plunger of the syringe is pushed forward, the fluid inside the syringe compresses, the walls of the syringe distend, and some of the fluid is expelled out of the syringe.

We define control volume as directly adjacent to the syringe plunger at time $t + dt$. With this definition, control volume is fixed in the frame of reference of the syringe. We then apply the

mass conservation equation [2]:

$$\int_{CV(t)} \frac{\partial \rho}{\partial t} dV + \int_{CS(t)} \rho v_n dA = 0 \tag{5}$$

where ρ is the density of the fluid inside the control volume, V is the volume of the control volume, v_n is the velocity component normal to control surface, $CV(t)$ is the control volume at time t and $CS(t)$ is the surface of the control volume at time t. Note that control surface is defined as the surface enclosing the control volume. This is a vector quantity directed along the outward normal to adjacent portion of the control volume.

The first term on the left hand side of eq. 5 simplifies to $V \cdot \frac{d\rho}{dt}$, since the control volume is defined to have a fixed volume. Applying a continuity boundary condition at all surfaces of the control surface, the second term on the left hand side simplifies to $\rho Q_{out} + \rho \frac{dV}{dt} - \rho A_0 \frac{dL}{dt}$. This simplifies eq. 5 to:

$$V \cdot \frac{d\rho}{dt} + \rho Q_{out} + \rho \frac{dV}{dt} - \rho A_0 \frac{dL}{dt} = 0 \tag{6}$$

In actuality, $\rho = \rho(P,T)$ where P and T are the pressure and temperature of the fluid respectively. Based on [3] and from the definition of bulk modulus β, we have $\beta = \rho_i \cdot \frac{\partial P}{\partial \rho}\Big|_{\rho_i, T_i}$ where ρ_i and T_i are the local density and temperature respectively at the location where the differential $\frac{\partial P}{\partial \rho}$ is evaluated. Then,

$$\dot{\rho} = \frac{\partial \rho}{\partial P} \cdot \frac{\partial P}{\partial t} = \frac{\rho_i}{\beta} \cdot \dot{P} \tag{7}$$

and eq. 6 simplifies to:

$$V \cdot \frac{\rho}{\beta} \cdot \dot{P} + \rho \cdot Q_{out} + \rho \dot{V} - \rho A_0 \dot{L} = 0 \tag{8}$$

Here ρ_i is replaced by ρ because, according to eq. 6, ρ is the local density of the fluid which is the same as ρ_i in eq. 7 at any given location.

We define syringe capacitance as the ratio of 'additional volume' stored in the syringe upon pressurization, represented by the functional form $\frac{A_0 \dot{L} - Q_{out}}{\dot{P}}$. Upon rearranging the terms in eq. 8, we get:

$$C_{S,tot} = \frac{A_0 \dot{L} - Q_{out}}{\dot{P}} = \frac{V}{\beta} + \frac{\dot{V}}{\dot{P}} \tag{9}$$

The term $\dot{V}/\dot{P}$ describes the capacitance as a result of the deformation of syringe walls when pressurized, and can be evaluated based on pressure vessel dynamics equations [4] as:

$$C_{S,w} = \frac{\dot{V}}{\dot{P}} = \frac{2V_S r(1 - \nu/2)}{Eb} \tag{10}$$

Where V_S is the volume enclosed within the syringe, r is the radius of the syringe, ν is the Poisson's ratio of the syringe material, E is the Young's modulus of the material and b is the thickness of the walls of the syringe.

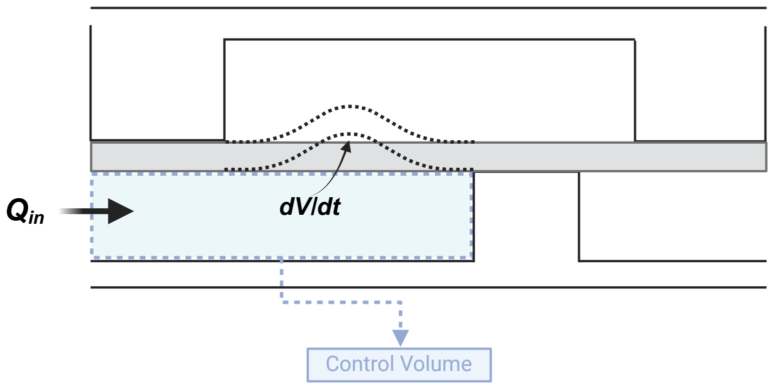

Figure S3: Valve capacitance schematic. As a result of the influx of fluid into the valve, the membrane distends and the fluid contained within the valve compresses.

S1.1.3 Valve Capacitance

We define the control volume as the volume enclosed underneath the membrane and connected to a syringe via microfluidic channel. Applying mass conservation eq. 5 to the valve, we get:

$$V\frac{d\rho}{dt} + \rho\frac{dV}{dt} - \rho Q_{in} = 0 \tag{11}$$

Applying the definition of bulk modulus from eq. 7, and defining $C_{valve} = Q_{in}/\dot{P}$ we get:

$$C_{V,tot} = \frac{V}{\beta} + \frac{\dot{V}}{\dot{P}} \tag{12}$$

The term $\dot{V}/\dot{P}$ describes the deformation of the membrane when the valve is pressurized, and has been evaluated previously [5,6] as:

$$C_{V,m} = \frac{\dot{V}}{\dot{P}} = \frac{6w^6(1-\nu^2)}{\pi^4 E t^3} \tag{13}$$

Where w is the width of the valve, ν is the material's Poisson's ratio, E is the modulus of elasticity, t is the thickness of the valve membrane.

In this model we have not included the permeability of fluid across membrane, because within the range of our operating parameters this has negligible impact on the full circuit simulation results, as described in section S3. In case the membrane permeability cannot be ignored, it can be modeled like in section S3 with appropriate permeability values evaluated experimentally.

S1.2 Full Circuit Model

S1.2.1 Pressurization

From the gas resistance definition in eq. 3, we can write flow rates at the syringe (Q_S) and valve (Q_V) ends of the resistor with liquid resistance R_0 as

$$Q_S = \frac{P_S^2 - P_V^2}{2P_S R_0}; \;\; Q_V = \frac{P_S^2 - P_V^2}{2P_V R_0} \tag{14}$$

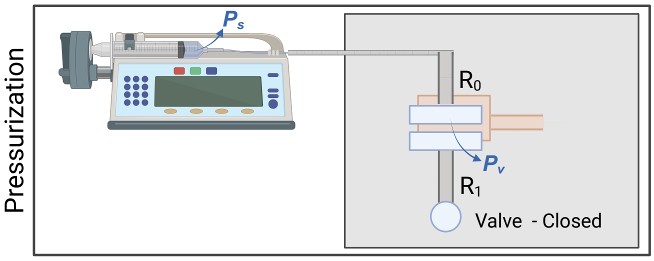

Figure S4: Full circuit schematic during valve pressurization. Since the valve is not open, the portion of the circuit between the syringe and the valve effectively behaves as a closed system.

In the syringe capacitance definition, we know from fig. 2B that the contribution from the syringe wall deformability towards total capacitance is negligible compared to that from the fluid compressibility. So, we can simplify the syringe capacitance as $C_{S,tot} = A_0\dot{L} - Q_{out}/\dot{P}$. Defining $A_0\dot{L}$ as Q_0, which is the flow rate set on the syringe pump, eq. 9 simplifies to:

$$\frac{Q_0 - Q_S}{\dot{P}_S} = \frac{V_S}{\beta}$$

For an isothermal process, bulk modulus β is equal to the local pressure, and the volume enclosed within the syringe at any given time t can be written as $A_0L_0 - Q_0t$, where L_0 is the total length of the syringe volume. Then, the above equation becomes:

$$\frac{dP_S}{dt} = P_S \cdot \frac{Q_0 - Q_{out}}{A_0L_0 - Q_0t}$$

Which, upon substituting for Q_S based on eq. 14, simplifies to

$$\frac{dP_S}{dt} = \frac{P_SQ_0}{A_0L_0 - Q_0t} - \frac{P_S^2 - P_V^2}{2R_0(A_0L_0 - Q_0t)} \tag{15}$$

From valve capacitance in eq. 12, we have

$$\dot{P}_V = (Q_V - \dot{V}_V) \times \frac{\beta}{V_V}$$

Where V_V is the volume enclosed underneath the valve membrane. Combining the above with eq. 14, we get

$$\frac{dP_V}{dt} = \left(\frac{P_S^2 - P_V^2}{2P_VR_0} - \dot{V}_V\right)\frac{P_V}{V_V}$$

which simplifies to

$$\frac{dP_V}{dt} = \frac{P_S^2 - P_V^2}{2R_0V_V} - \frac{\dot{V}_V \cdot P_V}{V_V}$$

Recalling that the capacitance contribution from the membrane deformability is defined as $\dot{V}_V = C_{V,m} \cdot \dot{P}_V$, this simplifies to

$$\frac{dP_V}{dt} = \frac{P_S^2 - P_V^2}{2R_0[V_V + C_{V,m} \cdot P_V]}$$

Substituting $V_V = V_{V,0} + C_{V,m} \cdot (P_V - P_{th,close})$ based on $C_{V,m}$ definition, we get

$$\frac{dP_V}{dt} = \frac{P_S^2 - P_V^2}{2R_0[V_{V,0} + C_{V,m}(2P_V - P_{th,close})]} \tag{16}$$

Equations 15 and 16 are solved to give the pressure-time relations for the syringe and valve respectively during the pressurization half of the cycle.

S1.2.2 Depressurization

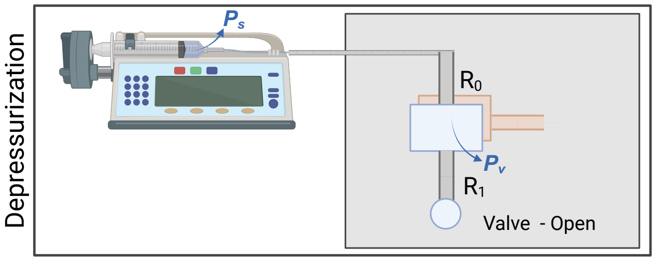

Figure S5: Full circuit schematic during valve depressurization. The valve vents to atmospheric pressure through resistor R_1 even as the syringe continues to pressurize the valve through R_0.

During depressurization, the syringe dynamics stay identical to the case of pressurization, so the syringe pressure equation is the same as eq. 15. Applying the control volume analysis to the valve, we get

$$Q_{in} - Q_{out} = \dot{V} + \frac{V}{\beta} \cdot \dot{P} \tag{17}$$

We can write the flow rates in terms of the pressure at either side of the valve and connecting resistances as:

$$Q_{in} = \frac{P_S^2 - P_V^2}{2P_V R_0}; \;\; Q_{out} = \frac{P_V^2 - P_{atm}^2}{2P_V R_1} \tag{18}$$

Given $C_{V,m} = \dot{V}_V / \dot{P}$ and $V_V = V_{V,0} + C_V, m(P_V - P_{th,open})$, eq. 17 and 18 combine to give

$$\frac{dP_V}{dt} = \frac{1}{V_{V,0} + C_{V,m}[2P_V - P_{th,open}]} \left(\frac{P_S^2 - P_V^2}{2R_0} - \frac{P_V^2 - P_{atm}^2}{2R_1} \right) \tag{19}$$

Equations 15 and 19 are solved to give the pressure-time relations for the syringe and the valve respectively during the depressurization half of the cycle.

S2 Simulation Results

S2.1 Capacitance Plots

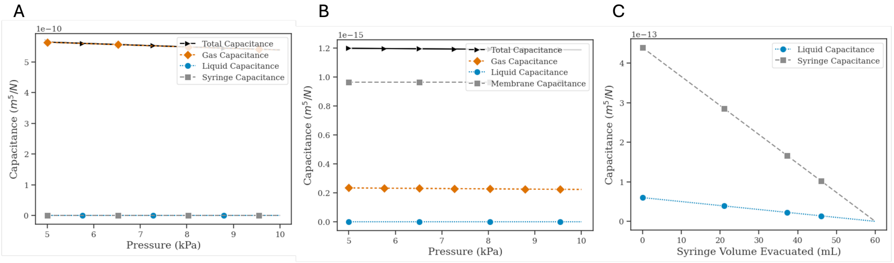

Figure S6: (A.) Total syringe capacitance, (B.) Total valve capacitance and their contributors; y axis on linear scale (C.) Syringe capacitance, liquid capacitance as syringe empties

Even though the syringe and the valve capacitance are functions of pressure, they do not change appreciably within the pressure range realized in the microfluidic device. Individual capacitance contributors and total capacitance for both elements of the microfluidic circuit are plotted on the linear scale in Fig. S6A, 6B against changing pressure to demonstrate the small changes in capacitance values with greater sensitivity. The X axis ranges from the threshold closing pressure to the threshold opening pressure.

Fig. S6C demonstrates that capacitance as a result of the syringe wall distension and the liquid compression also changes as the syringe empties, even though it is not visually evident in fig. 2C because gas compliance is much higher in comparison.

S2.2 Single cycle simulation results with different resistance values

Case 1: $R_0 = 5e11, R_1 = 4e13$

The valve fails to vent because the downstream resistance is too high. While the syringe pump

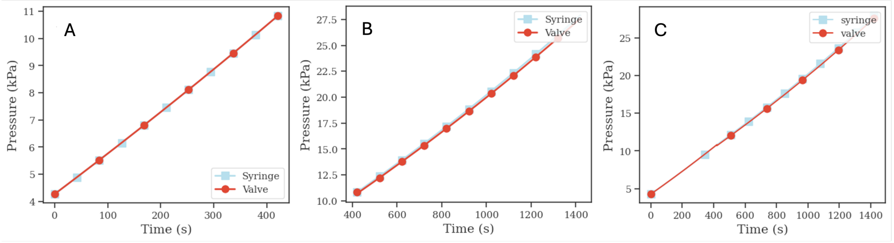

Figure S7: R_0 = 5e11, R_1 = 4e13 – (A.) Pressurization, (B.) depressurization and (C.) combined pressure graph.

stays on, pressure keeps on building up within the syringe and consequently in the valve.

Case 2: $R_0 = 5e11, R_1 = 4e12$

Even after a one order of magnitude reduction in the R_1 value, depressurization does not occur.

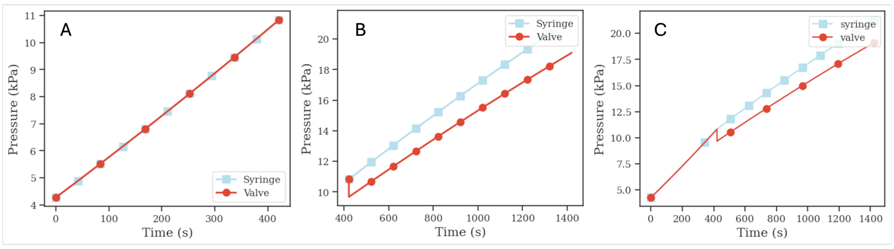

Figure S8: R_0 = 5e11, R_1 = 4e12 – (A.) Pressurization, (B.) depressurization and (C.) combined pressure graph.

Valve pressure briefly drops after the valve opens, but then the increase in pressure from the syringe overpowers the venting from the valve, and both the syringe and vale pressure increase with time.

Case 3: $R_0 = 5e11, R_1 = 4e11$

When R_1 is comparable to R_0, and in fact when R_1 is slightly smaller than R_0, the valve is able to

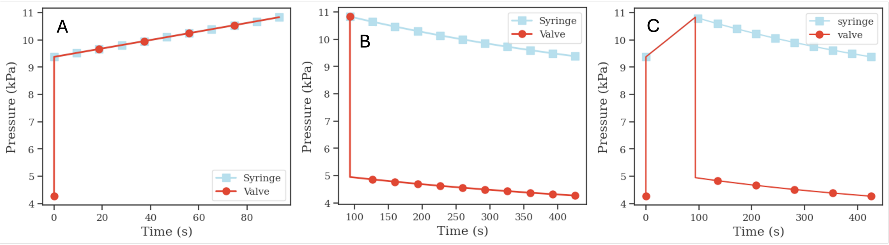

Figure S9: R_0 = 5e11, R_1 = 4e11 – (A.) Pressurization, (B.) depressurization and (C.) combined pressure graph.

successfully depressurize. Single cycle time period is also reasonable.

Case 4: $R_0 = 5e10, R_1 = 4e11$

However, R_1 is expected to be larger than R_0. So, we try reducing R0 while keeping R1 the same and see if we can get a realistic pressurization-depressurization curve. This oscillation cycle looks very similar to the one obtained from experiments. The time period also looks similar to experimental value. The estimated range for the resistance upstream of the valve is between 1e10 and 5e11, so these values are reasonable and are used for the other simulation results in the paper.

Discussion: It was found that when the downstream resistance is set two orders of magnitude higher than upstream resistance as in the case of the serpentine channel, the oscillator fails to discharge - in contrast to experimental results. Surprisingly, in similar computational models of liquid systems, the higher serpentine resistance was well tolerated but can be explained by the range of flow rates used. When a liquid working fluid is used to generate oscillatory flow

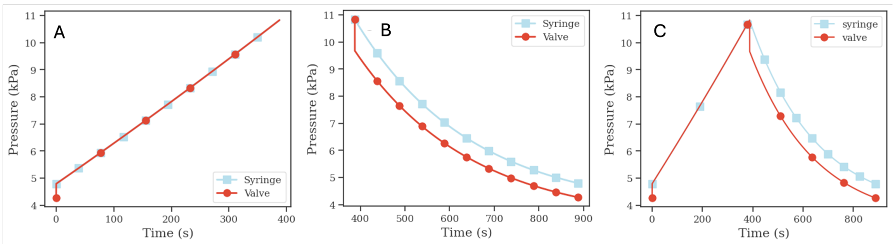

Figure S10: Figure 5: R_0 = 5e10, R_1 = 4e11 – (A.) Pressurization, (B.) depressurization and (C.) combined pressure graph after one oscillation cycle.

with the same microfluidic device design, the flow rate that is typically used is much lower (2-10 μL/min compared to 500 μL/min in case of gas). When the flow rate is very high, the rate of pressurization is correspondingly high. If the downstream resistance is also very high, then valve depressurization is constrained, and in some cases cannot match with the rate of pressurization, and the valve fails to discharge. This seems to indicate that our assumptions regarding the fluidic circuit discharge may be incorrect - the branch of the oscillator circuit connecting to the other valve cannot be ignored during valve discharge and must be considered in our model. Consequently, the effective drain resistance would be lower because the non-pressurized underside of the adjoining valve would behave as a capacitor and the (lower) resistor connecting the two valves would then act as the drain resistance.

S3 Membrane permeability to air

We can modify the model equations to include gas permeability through PDMS membrane to estimate its impact to over all results. When fluid leaks through the PDMS membrane, the control volume equation changes to

$$V\frac{d\rho}{dt}+\rho\frac{dV}{dt}-\rho Q_{in}+\rho Q_{out}=0 \tag{20}$$

This yields the valve equation:

$$\dot{P_V}=(Q_V-\dot{V_V})\times\frac{\beta}{V_V} \tag{21}$$

Substituting in the circuit equation, we get the following for the valve pressure during the pressurization half of the cycle.

$$\frac{dP_V}{dt}=\left[\frac{P_S^2-P_V^2}{2R_0}-Q_MP_V\right]\cdot\frac{1}{(V_{V,0}+C_{V,m}(2P_V-P_{th,close})} \tag{22}$$

From our experiments, we obtain a conservative estimate for the leakage flow rate as 3.84 μL/min. Substituting and running the simulations with the updated pressurization equation, we get the following plots:

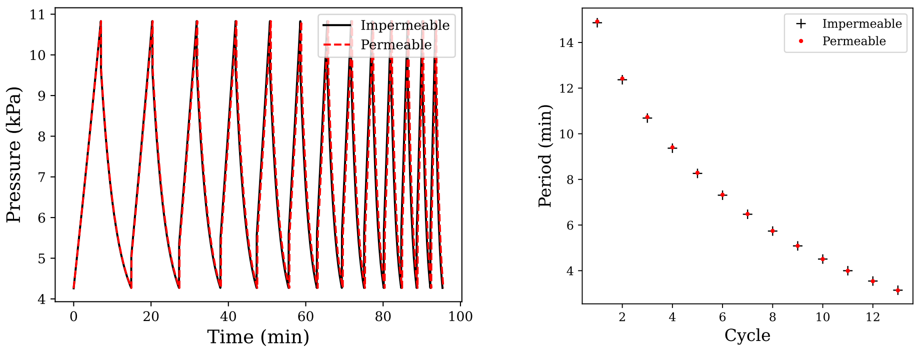

Figure S11: (A.) Pressure trace for one of the valves in the oscillator system over 13 pressurization-depressurization cycles and (B.) Time period over 13 oscillations, with (red) and without (black) gas permeability across PDMS membrane

We see from the updated figures that the pressure trace and the time period are nearly identical regardless of PDMS permeability. This is expected, because across roughly 100 mins of the oscillations shown here, of which about half the time accounts for pressurization of the valve, leakage accounts for a total of 192 μL, which is about 0.32% of the syringe volume. Looking through literature for the measurement of gas permeability through PDMS membranes, we find that the highest reported flow rate for a comparable pressure, membrane thickness and mixing ratio is around 4 μL/min [7,8,9], which is similar to our conservative measured value. Therefore, we can safely neglect gas permeability without a significant loss of accuracy.

Data to assess membrane permeability was collected by running the syringe pumps connected to the oscillators, as depicted in Figure 4 in the main text. However, instead of running the syringe pump continuously, both syringes were pushed to pressurize their respective valves, and before oscillations began between the two fluid lines, the syringe pump was stopped to assess the pressure drop across the closed valves over time. We assumed any pressure drop would be due to the gas loss from the permeability of PDMS, and that this would occur most quickly through the membrane. We chose to test oscillators with the thinnest membrane used for the study, a 20 μm membrane, presuming that this would lead to the most rapid loss due to the distance needed for gas diffusion across the PDMS to atmosphere. To calculate the membrane permeability, we calculated the leakage flow rate by taking the product of the difference in the channel's pressure over time and the total volume in the system once the syringe pump was stopped, and dividing it by product of the total time measured and atmospheric pressure. This yields a leakage flow rate equation for determining air loss through the PDMS system.

$$Leakage\ Rate = \frac{(P_f - P_i) \times (V)}{(t_f - t_i) \times (P_{atm})} \tag{23}$$

Where P_i and P_f are the initial and final pressures respectively, t_i and t_f are the initial and final time points respectively, V is the volume of our system, which we take to be the combined volume of the syringe, tubing and valve, and P_{atm} is the atmospheric pressure.

We assessed having both valves closed and captured both permeability rates simultaneously, in addition to having one valve open before we stopped the syringe. In the case of one valve being open, we would see the pressure decay for the open valve, while the other valve remained closed. Examples of data collected by the sensors of the syringes being pushed, the line and valves pressurizing, and a maintained pressure in the line is shown below in Figure S12. A total of 6 closed valves were measured. The average value of leak rate across closed valves was calculated to be 2.96 μL/min (standard deviation of 1.92 μL/min).

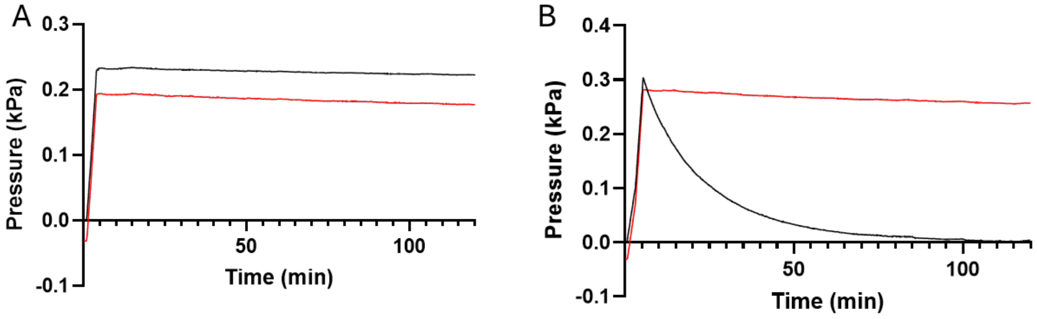

Figure S12: Pressure profiles of different forced air oscillators with one or two valves in a closed position to validate membrane permeability. Pressure data collected from two different 20 μm membrane devices are shown in the panels. The oscillator devices were drived by 60 mL syringes filled with air, which were run at 1,000 μL/min for 3 minutes before stopping the syringe pump. The pressure was monitored for at least 120 minutes to assess change in pressure. The oscillator in A) had both valves pressurized and in a closed position, reminiscent of pressurization prior to oscillations commencing for the first time. Leakage flow rates of these two closed valves was calculated to be 3.84 and 2.18 μL/min. The oscillator in B) had one valve open prior to stopping the pump, while the other valve remained in a closed state. The leakage flow rate from this oscillator was calculated to be 5.68 μL/min, the max leakage rate observed

S4 Experimental System

S4.1 Microfluidic Oscillator Pressure Profiles

Forced air microfluidic oscillators were evaluated over a range of flow-rate conditions, membrane thicknesses, and using different syringe sizes for the actuation of the devices. The figures provided below are meant to provide readers a broader array of demonstration that the microfluidic oscillators successfully oscillate to provide context for the readers that decreasing period is persistent across forced-air oscillations regardless of the conditions tested.

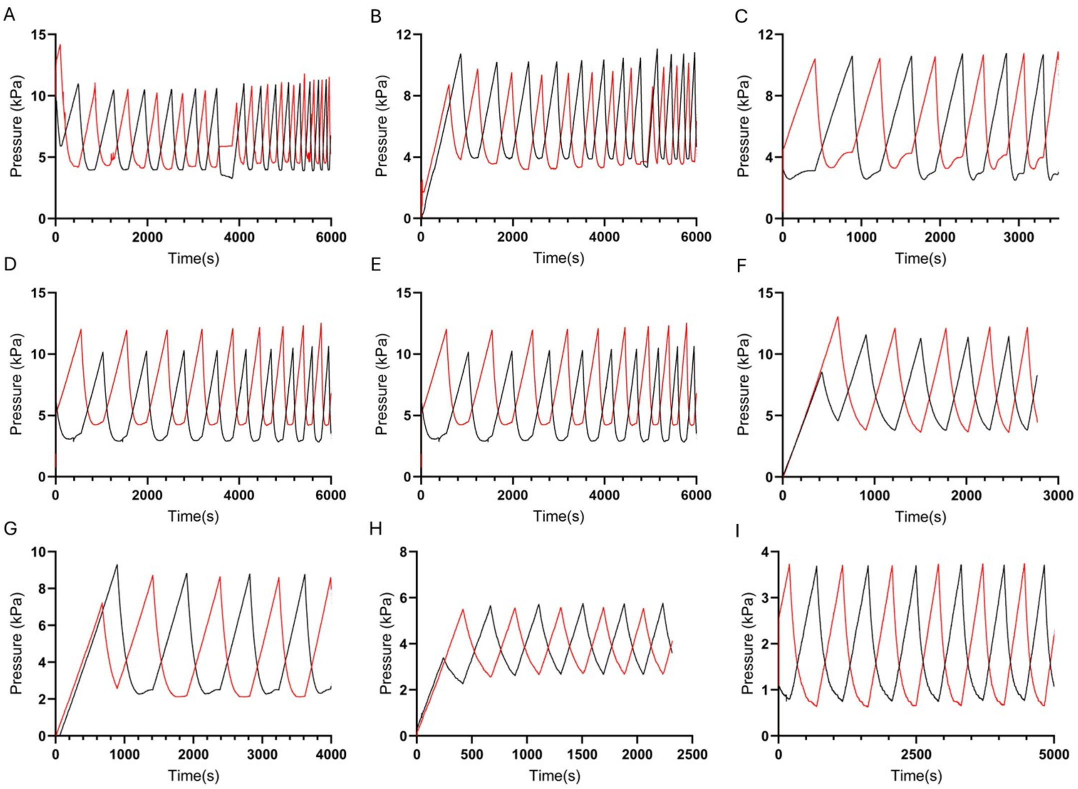

Figure S13: Pressure profiles of different forced air oscillators demonstrate oscillatory behavior across different membrane thicknesses, syringe sizes and flow rates. A) A 100 μm membrane device run at 500 μL/min ; B) a different 100 μm membrane device run at 500 μL/min; C) a 100 μm membrane device run at 400 μL/min; D) a different 100 μm membrane device run at 400 μL/min; E) a 70 μm membrane device run at 1000 μL/min; F) a 70 μm membrane device run at 750 μL/min (with 140 mL syringe); G) a 70 μm membrane device run at 400 μL/min; H) a 20 μm membrane device run at 500 μL/min; H) a 20 μm membrane device run at 200 μL/min. All syringes used for forced-air actuation of the microfluidic oscillators were 60 mL syringes unless otherwise noted.

S5 References

1. Duncan, P. N., Nguyen, T. V. & Hui, E. E. Pneumatic oscillator circuits for timing and control of integrated microfluidics. Proc. Natl. Acad. Sci. 110, 18104–18109 (2013).

2. Sonin, Ain A. "On Choosing and Using Control Volumes: Six Ways of Applying the Integral Mass Conservation Theorem to a Simple Problem," 2001. https://web.mit.edu/2.25/www/pdf/cv.pdf.

3. Ho, Nhut Tan. Chapter 5 Fluid Systems_part 1. https://www.ecs.csun.edu/ nhuttho/me584/ https://www.ecs.csun.edu/ nhuttho/me584/Chapter%205%20Fluid%20Systems_part%201.pdf.

4. Pressure Vessels – 3.11 Fall 1999 | Mechanics of Materials | Materials Science and Engineering. MIT OpenCourseWare https://ocw.mit.edu/courses/3-11-mechanics-of-materials-fall-1999/resources/mit3_11f99_pv/.

5. Bourouina, Tarik, and Jean-Paul Grandchamp. "Modeling Micropumps with Electrical Equivalent Networks." Journal of Micromechanics and Microengineering 6, no. 4 (December 1996): 398. https://doi.org/10.1088/0960-1317/6/4/006.

6. Zengerle, R., and M. Richter. "Simulation of Microfluid Systems." Journal of Micromechanics and Microengineering 4, no. 4 (December 1994): 192. https://doi.org/10.1088/0960-1317/4/4/004.

7. Eddings, Mark A., and Bruce K. Gale. "A PDMS-Based Gas Permeation Pump for on-Chip Fluid Handling in Microfluidic Devices." Journal of Micromechanics and Microengineering 16, no. 11 (September 2006): 2396. https://doi.org/10.1088/0960-1317/16/11/021.

8. Lamberti, A., S. L. Marasso, and M. Cocuzza. "PDMS Membranes with Tunable Gas Permeability for Microfluidic Applications." RSC Advances 4, no. 106 (2014): 61415–19. https://doi.org/10.1039/C4RA12934B.

9. Firpo, G., E. Angeli, L. Repetto, and U. Valbusa. "Permeability Thickness Dependence of Polydimethylsiloxane (PDMS) Membranes." Journal of Membrane Science 481 (May 1, 2015): 1–8. https://doi.org/10.1016/j.memsci.2014.12.043.
